# Functional consequences of archaic introgression and their impact on fitness

**DOI:** 10.1186/s13059-019-1920-z

**Published:** 2020-01-02

**Authors:** Maxime Rotival, Lluis Quintana-Murci

**Affiliations:** 1 Unit of Human Evolutionary Genetics, CNRS UMR2000, Institut Pasteur, 75015 Paris, France; 2 Chair Human Genomics & Evolution, Collège de France, 75005 Paris, France

Anatomically modern humans started to exit Africa for the first time at least 60,000 years ago (ya). Along their journey across the globe, they encountered and admixed with other hominins that are now extinct, such as the Neanderthals or Denisovans. Given the deep divergence time between ancient hominins and modern humans, such admixture events left molecular traces in non-African populations that are still visible today in their genomes [1]. Over the past few years, there is accumulating evidence to suggest that these segments of “archaic” DNA have the potential to contribute to phenotypic differences between contemporary individuals and populations [2]. Yet, to understand the genuine contribution of archaic alleles to the genetic architecture of complex traits, it is necessary to account for the diverse selective pressures that have acted upon introgressed alleles. Here, we discuss recent findings on how natural selection—either negative or positive—has shaped the landscape of Neanderthal ancestry in the genomes of modern Eurasians, and comment on the contribution of archaic haplotypes to present-day phenotypic variation.

## The cost of Neanderthal introgression

**Fig. 1:**
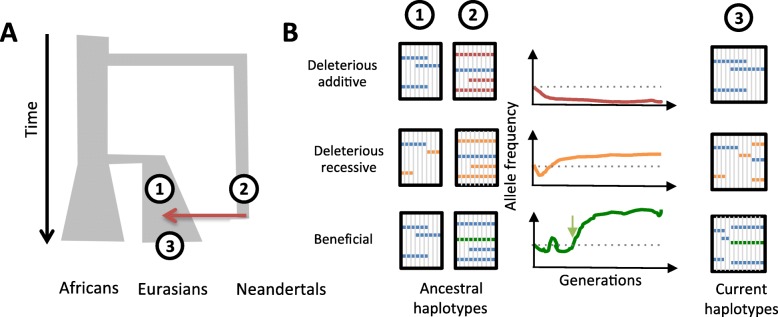
The fate of introgressed archaic haplotypes in the modern human genome. **a** Simplified demographic model of human populations. The size of the branches reflects effective population sizes (Ne), and a red arrow indicates Neanderthal introgression. Numbers indicate the relative position of the ancestral and present-day populations on the tree. **b** Haplotype structures and trajectory of archaic ancestry at three different regions that harbor distinct type of genetic variants (deleterious additive, deleterious recessive, beneficial). For ancestry trajectories, horizontal dotted line indicates the initial introgression frequency, green arrow represents the onset of selection for the beneficial allele. For haplotype structures, haplotypes are represented as columns. Neutral alleles are shown in blue, deleterious alleles in red (additive) or orange (recessive), and beneficial alleles in green

It has been suggested that the vast majority of alleles that Neanderthals contributed to modern humans were deleterious. The low genetic diversity of the available Neanderthal genomes indicates indeed that they had a limited effective population size, about 10-fold smaller than that of modern humans (Fig. 1a). Consequently, natural selection is expected to have been less efficient at removing deleterious mutations from the genome of Neanderthals than from the genome of modern humans [3]. Using forward simulations, Harris and Nielsen have shown that, prior to the admixture event(s), modern humans had higher fitness than Neanderthals, owing to a lower burden of deleterious alleles.

Assuming that the effect of deleterious mutations is mostly additive, they estimated that Neanderthal DNA was rapidly purged from the human genome after admixture, dropping from ~ 10 to the 2–3% currently observed in Eurasians [3] (Fig. 1b, upper panel). The purging was exacerbated in highly constrained regions, which exhibit decreased levels of Neanderthal ancestry. The rate of introgression is indeed strongly dependent on the intensity of background selection—a measure of the degree of linkage with regions that are highly conserved.

Conversely, in regions where most deleterious variants are recessive, Neanderthal ancestry may have actually been selected for [3] (Fig. 1b, middle panel). In these regions, a moderate rate of admixture confers a selective advantage to the admixed individuals, by increasing heterozygosity and decreasing the deleterious load. Further efforts are required to systematically quantify the deleteriousness of alleles that were present in the Neanderthal genome and the relative impact of recessive/additive variants on the fate of introgressed haplotypes. This, combined with measures of the local rate of human/Neanderthal divergence, will provide a better picture of the disparate landscape of Neanderthal ancestry along the genome of modern humans.

## The fate of introgressed functional alleles

Natural selection has had a profound impact on the landscape of archaic functional alleles that were introgressed. For example, Dannemann et al. have shown that non-synonymous archaic alleles that segregate today in the human population tend to be less deleterious than non-synonymous alleles that segregate at similar frequency on non-archaic haplotypes [4]. Furthermore, archaic introgression appears to be less pronounced in regions of functional relevance such as promoters or protein-coding regions, with respect to other elements such as enhancers [5, 6]. Given their larger size across the genome, enhancers are then the functional elements that carry the largest number of Neanderthal alleles [6]. It is therefore expected that a significant fraction of the phenotypic impact of Neanderthal introgression is mediated by changes in enhancer activity.

Despite the overall purge of archaic haplotypes in the genome of modern humans, Neanderthal haplotypes have been found to harbor more regulatory potential than their non-Neanderthal counterparts with similar allele frequency [4, 7]. This observation can be explained by an increased adaptive nature of Neanderthal haplotypes or, more simply, by the increase in local genetic diversity induced by the introgression event, owing to the high divergence between Neanderthals and modern humans. Massively parallel reporter assays, combined with deep learning approaches, may provide further insights into the mechanisms through which Neanderthal material, and specific genetic variants, affect human phenotypes.

## Epistatic incompatibilities of archaic functional alleles

To characterize the regulatory effects of archaic haplotypes, McCoy et al. have compared the relative expression of archaic and non-archaic alleles in a collection of 44 diverse tissues from the Gene–Tissue Expression database (GTEx) [8]. Neanderthal haplotypes tend to be generally biased towards lower expression levels, this effect being most pronounced in the brain and testis. This observation has been interpreted as supporting the occurrence of genetic incompatibilities between Neanderthals and modern humans, due to epistatic interactions as predicted by the Dobzhansky–Muller model of speciation (i.e., the fixation of incompatible mutations in two offspring lineages that share a common parental lineage). A weaker archaic ancestry on the X chromosome and near testis-expressed genes supports further the notion of a high rate of infertility among first-generation hybrids [1].

The enrichment in archaic haplotypes among loci that are associated with neurological and psychiatric disorders [2], together with the lower expression of archaic haplotypes in the brain, suggests that epistatic effects also affected cognitive capacities in hybrid individuals. Further work is clearly needed to assess the contribution of epistatic incompatibilities to the purging of functional Neanderthal alleles from the human lineage.

## The adaptive nature of introgressed DNA

Despite the overall deleteriousness of Neanderthal material in the genomes of modern humans [5], it is increasingly accepted that, in some cases, archaic DNA allowed early Eurasians to adapt to their newly encountered environments (Fig. 1b, lower panel). Detecting these events of adaptive introgression remains a daunting task, as the signatures used to detect positive selection (e.g., extended haplotype homozygosity) are similar to those left by archaic introgression, leading to spurious signals. To efficiently capture the adaptive nature of introgression, Racimo et al. have proposed a statistical framework based on the number and allelic frequencies of sites that are uniquely shared between archaic hominins and specific modern populations [9].

Using this framework, multiple genomic regions presenting compelling evidence of adaptive introgression have been detected [9], including regions associated to skin pigmentation or response to UV radiation and genes such as *BNC2*, *POU2F3*, or *HYAL3*. Metabolic processes have also been found as targets of adaptive introgression, including genes such as *SLC16A11*, known to alter lipid metabolism and type 2 diabetes risk, or *TBX15/WARS2*, associated to adipose tissue differentiation and body fat distribution.

Importantly, immune functions appear to be privileged targets of adaptive introgression, suggesting that modern humans acquired from Neanderthal adaptive variants related to host survival against infection. Evidence supporting this notion has been reported for the Toll-like receptor *TLR1/6/10* cluster, primarily involved in the sensing of bacterial products, and for several antiviral response genes, such as the NOD-like receptor *NLRC5*, the cytoplasmic sensor *IFIH1*, or the restriction factors *OAS1/OAS3*. It is interesting to note that an excess of regulatory variants (i.e., eQTLs) controlling transcriptional responses to viral stimuli has also been reported among Neanderthal haplotypes, with respect to non-archaic haplotypes [7].

Consistent with these results, Enard and Petrov have shown that adaptive introgression from Neanderthals has been pervasive among human virus-interacting proteins (VIPs), the strongest enrichment being observed for VIPs interacting with RNA viruses [10]. These results collectively emphasize the important role of introgression in human adaptation, in particular to pathogen pressures. Yet, new methods are needed to characterize how subtle but coordinated shifts in frequency of archaic haplotypes have contributed to modern human adaptation involving polygenic traits.

## Recovering lost genetic diversity through archaic admixture

The phenotypic impact of adaptively introgressed haplotypes is mediated, in some cases, by genetic variants that are not of Neanderthal origin themselves. This is notably the case for the well-characterized *OAS1* locus [11]. The rs10774671-G allele, which is present in Europeans specifically on Neanderthal haplotypes, alters the splicing patterns of *OAS1*, leading to increased anti-viral activity. Interestingly, this variant is also present at high frequency in African populations, where it lies on a distinct haplotypic background. These observations suggest that an introgression event occurring in Eurasians and targeting the *OAS1* locus re-introduced a beneficial allele that had been lost during the out-of-Africa bottleneck.

Recent work by Rinker et al. suggests that the re-introduction through introgression of ancient functional alleles—i.e., predating the split between Neanderthals and modern humans—has been a common phenomenon [12]. Yet, the extent to which such re-introduced variants have contributed to the adaptive nature of archaic introgression remains an open question.

## Beyond Neanderthal: archaic introgression from other hominins

Most efforts in understanding the functional consequences of archaic introgression have been focused on Neanderthals and modern Eurasians, primarily of European ancestry. However, the number of ancient genomes is increasing, including high coverage whole-genomes from various Neanderthals (Altai, Vindija, and Chagyrskaya) and the Denisovan Altai. This, together with the possibility of identifying segments of archaic DNA directly from modern genomes, clearly opens a highly informative window to study the patterns of population diversity of ancient, now-extinct hominins and their admixture history with modern humans.

Dissecting and quantifying the archaic ancestry in the genomes of modern Oceanians, for example, offer an incredible access to the past history of Denisovans and the extent to which they contributed to the adaptation of early modern humans entering the Pacific. Likewise, the possibility that early Africans also admixed with a yet-unknown ancient hominin is increasingly supported but needs further investigation, both methodological and empirical. We have exciting times in front of us that, all together, will provide a much finer understanding of the functional consequences of archaic introgression in modern humans, their adaptive nature and their contribution to the diversity of human phenotypes.
